# Supplementary material for: Exploring loneliness: Prevalence and determinants among medical students in Bangladesh
Source: PLOS Ment Health. 2025 Aug 20;2(8):e0000379. doi: 10.1371/journal.pmen.0000379 (PMC12798429; doi:10.1371/journal.pmen.0000379)
Supplement: Attachment — Submitted filename: Response to Reviewers.docx [file pmen.0000379.s003.docx]

**Response to Reviewers and Editor**

*Manuscript ID: PMEN-D-25-00014*

*Title: Exploring loneliness: Prevalence and determinants among medical students in Bangladesh*

Dear Editor and Reviewers,

We thank you sincerely for the opportunity to revise and resubmit our manuscript to PLOS Mental Health. We are grateful for the insightful comments and constructive feedback provided by the editor and reviewers, which have significantly helped us improve the quality, clarity, and rigor of our work. Below, we provide detailed responses to each of the comments raised.

We have addressed and revised the manuscript accordingly and highlighted all changes in the revised version for easy reference.

**EDITOR'S COMMENTS**

1. **Data Sharing:**

**Comment:** I agree with the reviewers that anonymized data should be possible to share. The authors should consider doing this and check with their ethics committee/institutional review board how they can go about it.

**Response:** We appreciate this important point. A Data Availability Statement to the manuscript indicating that the anonymized data will be made available upon publication.

**2. Statistical Issues:**

**Comment:** Once again, I agree with the reviewers that the work requires more careful statistical analyses and grounded language around inference.

**Response:** Thank you for highlighting this important concern. We fully acknowledge the need for more rigorous statistical analysis and cautious language around inference. As our study employed only basic descriptive and bivariate analyses, we have now clearly stated this in the Limitations section. Specifically, we have acknowledged the absence of multivariate analyses, effect size reporting, and tests for normality, which may limit the strength of the inferences drawn.

We have also revised the manuscript’s discussion and conclusion sections to ensure our language reflects associations rather than causal interpretations, in line with the statistical limitations of the study.

We sincerely appreciate the guidance, which has helped us improve the clarity, transparency, and academic rigor of the manuscript.

**REVIEWER'S COMMENTS**

**Reviewer #1**

Reviewer #1: I enjoyed reading the manuscript and you made some very interesting observations.

Response: Thanks for your kind comment. We really appreciate your time to read our manuscript thoroughly and provide very useful feedback.

There are several underlying issues that if addressed could bring additional clarity to the article:

1. There needs to be a clear definition of loneliness. This is critical to understand the rest of the paper. Without a working definition and parameters of this at the beginning, the understanding of the rest of the paper is limited.

**Response:** Thank you for highlighting this important point. In the revised manuscript, we have now added a conceptual definition of loneliness in the Introduction section at 100^th^ line as follows:

Loneliness is the subjective feeling of isolation, lack of belonging, or absence of meaningful companionship. It is recognized as an emotional and mental state that may manifest as psychological distress, including sadness, low self-esteem, and hopelessness, particularly when individuals perceive a gap between their desired and actual social connections [25, 26].

2. There needs to be additional explanations regarding the statistical analysis used when considering the sociodemographic characteristics. You report significant findings, but without enough clarity to fully understand.

**Response:** Thank you for your insightful comment. We acknowledge that the statistical analysis section lacked sufficient explanation and clarity. As the study employed only basic descriptive and bivariate analyses (e.g., chi-square tests and t-tests), we did not perform multivariate analysis to adjust for potential confounders. We recognize this as a significant limitation. We have added the following to the Limitations section:

The study did not include multivariate analysis to control for confounding variables, nor did it calculate effect sizes or test for normality assumptions prior to conducting t-tests. This limits the ability to infer independent associations and may affect the robustness and generalizability of the findings. Future studies with more rigorous statistical methods are recommended to validate these associations.

3. On line 173 your title is prevalence of depression, but this is inappropriate because it is not what you are measuring.

**Response:** Thank you for this observation. We corrected this unintentional mistake accordingly.

4. There needs to be more comparisons to the general population of Bangladesh. You make comparisons with some socio demographic info, but it would be nice to see comparisons in loneliness (if they exist)

**Response:** Thank you for this thoughtful suggestion. After an extensive literature search, we found that there is currently no nationally representative dataset that directly reports the prevalence of loneliness in the general population of Bangladesh. The National Mental Health Survey 2018–2019, conducted by the National Institute of Mental Health (NIMH) and the World Health Organization (WHO) Bangladesh, is the most comprehensive source of mental health data. While it does not include loneliness as a specific variable, it does report that 6.7% of adults suffer from depressive disorders and 4.5% from anxiety disorders, both of which are often linked with loneliness. To bridge this gap, our study contributes to the growing body of evidence by providing data on loneliness among a specific and high-risk subgroup—medical students. We have added additional papers to compare the general population of Bangladesh as suggested by you in the Discussion section of the revised manuscript as follows:

Loneliness is a significant concern across different demographic groups, with varying prevalence rates reported depending on age, setting, and context. Among graduate university students, a study conducted in Bangladesh reported that approximately 43% experienced loneliness, indicating a substantial mental health burden even among young and educated populations [27]. In contrast, older adults tend to report even higher levels of loneliness. One international study that included individuals aged 60 years and above found that more than half (51.5%) of participants experienced loneliness, highlighting the emotional vulnerability of aging populations [28]. Similarly, a longitudinal survey analyzing loneliness trends during the COVID-19 pandemic revealed a high baseline prevalence of 51.5% in 2020, which declined modestly to 45.7% in 2021, though nearly half of the respondents continued to report loneliness, suggesting persistent challenges despite temporal improvements [29].

Focusing on the context of Bangladesh, a community-based study among older adults found the prevalence of loneliness to be 54.3%, indicating that loneliness among elderly individuals in Bangladeshi societies may be particularly pronounced due to factors such as limited social support, declining health, and intergenerational separation [30]. These findings collectively demonstrate that loneliness is not restricted to one particular age group or setting, but rather presents a widespread public health issue. The consistently high prevalence across studies underscores the need for context-specific interventions aimed at reducing social isolation and enhancing emotional well-being across the life course.

**Reviewer #2**

This study addresses an important and timely topic in mental health research. It highlights the high prevalence of loneliness among medical students and the association with various sociodemographic and behavioral risk factors.

Moreover, this study is well-structured and provides valuable insights. However, several methodological, statistical, and language-related concerns need to be addressed before the manuscript can be considered for publication in PLOS Mental Health. I encourage the authors to carefully revise their manuscript to enhance its impact and credibility. The statistical analysis is not fully rigorous due to the lack of multivariate analysis to control for Confounders variables; there is missing the effect sizes and unverified normality of the assumptions. Author needs to ensure the effect sizes, the OR and CI reported. Also, check the normality assumptions before performing the t-test. There is a lack of data availability statement due to ethical concerns but this needs to be requested. The Conclusion and implication of this study need to imply causal relationships rather than associations, lack of discussion on gender differences in loneliness, author need to suggest specific interventions to address loneliness among medical students.

**Response:** Thanks for your wonderful suggestions which we believe will be immensely useful to amend the manuscript. Please find our specific responses as follows:

1. Lack of multivariate analysis, control for confounders, normality assumptions and effect sizes:

Response: We appreciate this important observation. Due to the exploratory nature of our cross-sectional study and resource limitations, we did not conduct multivariate analysis to control for potential confounding variables, nor did we calculate effect sizes. We acknowledge this as a methodological limitation and have addressed it clearly in the revised manuscript under the Limitations section as follows:

The study did not include multivariate analysis to control for confounding variables, nor did it calculate effect sizes or test for normality assumptions prior to conducting t-tests. This limits the ability to infer independent associations and may affect the robustness and generalizability of the findings. Future studies with more rigorous statistical methods are recommended to validate these associations.

3. Data availability and ethics:

**Response:** We appreciate this important point. A Data Availability Statement to the manuscript indicating that the anonymized data will be made available upon publication.

4. Causal language in conclusions:

Response: Thanks for this feedback. We now amended the conclusion with academic language avoiding the casual tone as suggested as follows:

This study identifies a high prevalence of loneliness among medical students in Bangladesh and underscores its association with a range of socio-demographic and lifestyle factors. The findings contribute to the growing body of literature on mental health concerns in medical education and offer critical insights into the psychological vulnerabilities of this population. Nevertheless, certain methodological limitations should be acknowledged, including the reliance on online data collection and the use of convenience sampling, which may affect the generalizability of the results. Despite these constraints, the study underscores the pressing need for comprehensive mental health support mechanisms within medical institutions in Bangladesh. Evidence-based interventions—such as accessible counseling services, structured stress management programs, and the incorporation of mental health literacy into the medical curriculum—should be prioritized to enhance the psychological well-being and academic performance of medical students, ultimately contributing to a more resilient future healthcare workforce.

5. Gender differences and intervention recommendations:

**Response:** Thanks so much for this. We amended the recommendation section as follows, Regular monitoring and the implementation of tailored interventions that address gender-specific needs are essential to effectively support the evolving mental health challenges faced by medical students.

We did not propose any specific intervention; instead, we emphasized the importance of identifying the needs and priorities of medical students in order to design and implement appropriate, evidence-based interventions for them

**Reviewer #3**

The study demonstrates a technically sound approach with appropriate methodologies except using non-probability sampling which affect generalizability. The conclusion reports that 56% of participants experienced moderate to high levels of loneliness and the various socio-demographic and lifestyle factors associated with loneliness which is supported by the findings of the study.

**Response:** Thanks so much for your very insightful comment. We mentioned the use of non-probability sampling in the limitation section as follows: Consequently, the findings should be interpreted with caution, particularly given the convenience sampling techniques, limited sample size and potential lack of broader representation.

We hope that the revised manuscript addresses all the concerns raised. Thank you once again for the opportunity to improve our manuscript and contribute to this important field.

Sincerely,

Dr. Zarin Tasnim Maliha

On behalf of all co-authors
